# Recombinant human Bone Morphogenetic Protein-2 (rhBMP-2) in posterolateral lumbar spine fusion: complications in the elderly

**DOI:** 10.1186/1749-799X-8-1

**Published:** 2013-01-14

**Authors:** Martin F Hoffmann, Clifford B Jones, Debra L Sietsema

**Affiliations:** 1 Grand Rapids Medical Education Partners, 1000 Monroe Ave NW, Grand Rapids, MI 49503, USA

## Abstract

**Study design:**

Retrospective cohort study of 1430 patients undergoing lumbar spinal fusion from 2002 - 2009. Objective: The goal of this study was to compare and evaluate the number of complications requiring reoperation in elderly versus younger patients.

**Summary of background data:**

rhBMP-2 has been utilized off label for instrumented lumbar posterolateral fusions for many years. Many series have demonstrated predictable healing rates and reoperations. Varying complication rates in elderly patients have been reported.

**Materials and methods:**

All patients undergoing instrumented lumbar posterolateral fusion of ≤ 3 levels consenting to utilization of rhBMP-2 were retrospectively evaluated. Patient demographics, body mass index, comorbidities, number of levels, associated interbody fusion, and types of bone void filler were analyzed. The age of patients were divided into less than 65 and greater than or equal to 65 years. Complications related to the performed procedure were recorded.

**Results:**

After exclusions, 482 consecutive patients were evaluated with 42.1% males and 57.9% females. Average age was 62 years with 250 (51.9%) < 65 and 232 (48.1%) ≥ 65 years. Patients ≥ 65 years of age stayed longer (5.0 days) in the hospital than younger patients (4.5 days) (p=0.005).

Complications requiring reoperation were: acute seroma formation requiring decompression 15/482, 3.1%, bone overgrowth 4/482, 0.8%, infection requiring debridement 11/482, 2.3%, and revision fusion for symptomatic nonunion 18/482, 3.7%. No significant differences in complications were diagnosed between the two age groups. Statistical differences were noted between the age groups for medical comorbidities and surgical procedures. Patients older than 65 years underwent longer fusions (2.1 versus 1.7 levels, p=0.001).

**Discussion:**

Despite being older and having more comorbidities, elderly patients have similar complication and reoperation rates compared to younger healthier patients undergoing instrumented lumbar decompression fusions with rhBMP-2.

## Introduction

In the United States, improvements in health have resulted in increased life expectancy and contributed to the growth of the older population over the past century. As in most western countries, U.S. demographics show a graying trend over the last 30 years. In 1980, 11.31% of the U.S. population were 65 years or older [1]. In 2010, 12.97% of the US population were 65 years or older with an estimated increase to 20% in the following 30 years [1]. Today’s elderly are better educated and make higher demands for mobility and quality of life [2]. Therefore, the number of elderly patients presenting with degenerative disease of the lumbar spine qualifying and requiring surgery is expected to increase. In addition, elderly patients frequently have lumbar stenosis treated with extensive decompressive laminectomy and subsequent arthrodesis [3]. Instrumented posterolateral fusion is the most common type of fusion performed in the lumbar spine. Obtaining and utilizing autologous iliac crest bone for spinal arthrodesis is currently considered the “gold standard” [4]. Advanced patient age, medical comorbidities, and declining bone quality of the vertebra and iliac crest are important issues to consider when selecting the graft treatment for lumbar spinal fusion in older patients [5].

Degenerative lumbar spine diseases usually occur at multiple levels and require a greater amount of bone graft material [6]. Bone quality and bone availability are reduced in elderly patients. Potential alternatives and enhancements to autologous bone graft have gained attention. A wide array of bone graft materials is used to obtain solid fusion. The discovery of osteoinduction through demineralized bone protein extracts and later identification of bone morphogenetic proteins (BMPs) [7,8] offers the potential to obtain fusion without iliac crest bone graft harvesting or enhances the potential of limited autologous bone grafts.

Recombinant human BMP-2 (rhBMP-2) is a growth factor that belongs to the transforming growth factor-β (TGF–β) superfamily. rhBMP-2 (INFUSE, Medtronic, Memphis, TN) is commercially available for clinical use. Clinical use of rhBMP-2 was approved by the U.S. Food and Drug Administration (FDA) in 2002 for anterior lumbar spine fusions from lumbar level four (L4) to sacral one (S1) [9] and for the repair of symptomatic, posterolateral lumbar spine pseudarthrosis [10]. Despite this fact, off-label use of rhBMP-2 in combination with iliac crest autograft and/or bone void fillers for posterolateral intertransverse process fusion has become immensely popular because of the need for larger bone graft amounts required for this procedure especially in patients with poor and limited bone quality. The use of rhBMP-2 in posterolateral lumbar fusions is intended to enhance fusion mass and reduce donor side morbidity related to bone graft harvest [11] as described by Boden [12]. Singh [13] showed enhanced fusion rates for iliac crest bone graft in combination with rhBMP-2 compared to iliac crest bone graft alone. Following these promising results, rhBMP-2 utilization nationwide has increased to 28.1% of all fusion procedures [14].

Increasing age has been found as a risk factor for the development of postoperative complications in posterior lumbar decompression and arthrodesis [15]. Therefore, BMP supplementation might be the solution to achieve reliable fusion rates in elderly patients. In contradiction, a recent report noted reduced fusion rates with rhBMP-2 among adults over 65 years of age, which was attributed to weakened osteoinductive capacity in elderly patients [6]. In addition, multiple complications attributed to BMPs have been observed [16,17]. According to our knowledge, no correlation of these BMP specific complications to age has been published. We assume that there is a higher complication rate in older patients and in patients with comorbidities. BMP related complications are uncommon and therefore a large study population is necessary to determine complication rates.

The actual data is still lacking to determine an acceptable safety profile for rhBMP-2 in posterolateral fusions for older patients. The purpose of this retrospective cohort was to determine whether there was a difference in the number of complications requiring reoperation in elderly versus younger patients.

## Materials and methods

This study was an Institutional Review Board approved retrospective cohort exploratory review of patients undergoing instrumented lumbar posterolateral fusion utilizing of rhBMP-2 (INFUSE) at a single private practice associated with a tertiary referral spine center. It was approved by the Spectrum Health Institutional Review Board, Grand Rapids, Michigan. The IRB study # was 2007-123. Five fellowship trained orthopaedic surgeons performed all operative procedures. Consecutive patients were identified by Current Procedural Treatment (CPT) codes for fusion (22612, 22614), instrumentation (22840, 22842, and 22843) with corresponding rhBMP insertion that had initial treatment from March 2002 through June 2009, 7 years. Inclusion criteria were: instrumented lumbar posterolateral fusion of one to three levels with rhBMP-2 implant and age equal to or older than eighteen years. Exclusion criteria were: thoracic fusions, additional anterior, posterior, or transforaminal lumbar interbody fusion (ALIF, PLIF, TLIF), BMP products other than rhBMP-2, follow up less than 6 months, and insufficient medical record or radiographic data.

Of a total of 2406 lumbar fusions performed during the study period, 598 patients underwent isolated instrumented lumbar posterolateral fusion of ≤ 3 levels with the addition of rhBMP-2. One hundred and sixteen (116) patients were excluded because of death during hospitalized or postoperative period (2), utilization of additional bone stimulator (9), and inadequate follow up data (105). One death during hospitalization occurred as a result of pulmonary embolism and one death occurred four weeks after discharge not related to the surgical procedure. 482 patients were identified with a mean age of 62 years (range 18–89). There were 203 (42.1%) males and 279 (57.9%) females. Average body mass index (BMI) was 31.4 (18.4-63.9) kg/m^2^.

In all patients, a trail of non-operative treatment including non-steroidal anti-inflammatory drugs (NSAID), physical therapy, and/or spinal injections was ineffective. 395 patients had preoperative back pain. 325 patients had preoperative radicular leg pain. All patients had neurogenic claudication. Surgery indications are listed in Table 1.

**Table 1:** Indications for decompression and lumbar fusion

**Indications for surgery(multiple reasons possible)**	**Number**	**Percentage**
Back pain	395	82.0%
Radicular leg pain	325	67.4%
Degenerative disc	33	6.8%
Spondylolisthesis	228	47.3%
Instability	173	35.9%
Spondylosis	70	14.5%
Stenosis	430	89.2%
Failed previous fusion	18	3.7%

Diagnosis, spinal pathology, and surgeon’s best knowledge determined treatment. Therefore in total, 899 levels were fused. Number of levels fused was: 1 (153, 31.7%), 2 (241, 50.0%), and 3 (88, 18.3%). Bone graft and bone graft extenders were used at the surgeons’ discretion. All patients underwent local autografting utilizing decompressed posterior elements.

All patients were consented to lumbar decompression and arthrodesis utilizing rhBMP with education and information of the off label utilization of rhBMP-2. All patients received antibiotics (cefazolin) 30 minutes pre- and 24 hours post-operatively. Some patients had preoperative and intraoperative steroids. Steroids were utilized based upon patient symptoms, surgeon discretion, and anesthesiologist experience. The posterolateral arthrodesis was prepared with decortication of the posterior aspect of the transverse processes with a high-speed burr. The posterior elements were denuded of soft tissue or cartilaginous attachments, ground into small fragments, and placed onto the transverse processes. The rhBMP-2 was prepared according to manufacturer specifications (Medtronic, Memphis, TN). The rhBMP-2 attached to the bovine collagen sponge was wrapped around a bone void filler (BVF) such as Mastergraft (Medtronic, Memphis, TN) or allograft bone (Musculoskeletal Transplant Foundation, MTF, West Chester, PA) and placed onto the transverse processes and posterior elements. The sponge required proximity and contact with all transverse processes of the levels undergoing arthrodesis. The surgeon determined the amount or number of INFUSE kits utilized per surgery. The mean total BMP-2 dose implanted was 12.4 mg per patient (range 12.0-24.0). The mean dosage per level was 7.7 mg (range 4.0-24.0). Once the procedure was completed, the fascia was closed over a hemovac drain. The surgeon determined the timing of drain removal.

Patient follow-up was performed at regular intervals of two weeks, six weeks, twelve weeks, six months, one year, and later if required or indicated. The mean follow up period was 15.8 months (range 6–70 months). Clinical examination, pain scales, and radiographic imaging were performed at these intervals. Lumbar radiographs consisted of anterior-posterior (AP) and lateral (Lat) views. Flexion/extension radiographs or CT imaging was performed to confirm arthrodesis, screw loosening, instability, nonunion, ectopic bone formation, and/or cortical erosion. MRI with gadolinium of the lumbar spine was obtained if neural symptoms or pain indicated. Complications related to the performed procedure were recorded. Additional or revision surgeries were performed when indicated.

Patients were grouped by age into Group 1: 18–64 years, Group 2: ≥ 65 years. Comorbidities and potential contributing factors were recorded (Table 2). Descriptive statistics were completed. Chi square and t-tests were used to compare age groups less than 65 and those greater than or equal to 65 for demographic data, complications contributing factors, and rhBMP-2 used. Data was analyzed using PASW/SPSS® 18 (IBM, New York, NY).

**Table 2:** Risk factor differences in age groups

**Contributing factors**	**Group 1**	**Group 2**	**Significance**
	**(18-64 years)**	**(≥65 years)**	
	**N=250**	**N=232**	
**Gender**			χ²=0.292
**male**	111 (44.4%)	92 (39.7%)	
**female**	139 (55.6%)	140 (60.3%)	
**BMI(kg/m²)**	32.2^a^ (18.4-63.9)	30.5 (18.5-57.2)	p=0.006
**Diabetes**	35 (14.0%)	49 (21.1%)	χ²=0.109
**Cardiovascular disease**	114 (45.6%)	147 (63.4%)	χ²<0.001
**Respiratory disease**	58 (23.2%)	40 (17.2%)	χ²=0.104
**Current Tobacco Use**	51 (20.4%)	6 (2.6%)	χ²<0.001
**Allergies**	121 (52.6%)	97 (46.0%)	χ²=0.234
**Steroid medication**	53 (21.2%)	68 (29.3%)	χ²=0.081
**Preoperative NSAID**	83 (37.7%)	107 (46.1%)	χ²=0.010
**Previous BMP exposure**	10 (4.0%)	8 (3.4%)	χ²=0.740

BMI=Body Mass Index, BMP=Bone morphogenetic protein, NSAID= non-steroidal anti-inflammatory drugs.

## Results

Mean length of hospital stay (LOS) was 4.7 days (range 2–21 days) for all age groups combined. Patients ≥ 65 years of age stayed longer (mean 5.0 days, range 2–21) in the hospital than younger patients (mean 4.5 days, range 2–12) (t=0.757, p=0.005). LOS was not affected by the outlier of 21 days. In the <65 years group, 57% of the patients had a LOS of 3 and 4 days. In the ≥ 65 years group, 55% of the patients stayed in hospital for 4 and 5 days. No significant difference in length of stay between patients with (4.75 ± 1.9 days) or without additional iliac crest bone grafting (4.42 ± 1.8 days) was noted (t = 0.966, p=0.335).

Fifty patients of the 482 (10.4%) required repeat surgery due to surgical complications, with more than one complication occurring in some patients. Eleven patients had an intraoperatively diagnosed and repaired dural tear (2.3%). Complications requiring reoperation were: 18 of 482 (3.7%) patients with a symptomatic nonunion, 15 of 482 (3.1%) patients with a painful perioperative seroma formation required decompression with 5 positive intraoperative cultures (2 staphylococcus species, 1 methicillin resistant staphylococcus aureus, 2 propionibacterium species), 15 of 482 (3.1%) patients with excess bone reformation of which four required operative decompression (0.8%), 11 of 482 (2.3%) patients with infection, and one patient (0.2%) with malpositioned implant.

Mean age was 62 years (range 18–89) with an almost even distribution between less than 65 years (250, 51.9%) and greater than or equal to 65 years (232, 48.1%). Gender distribution was similar between the two groups with more female patients in the older age group (60.3% versus 55.6%; χ^2^=0.292). Younger patients had a greater body mass index (BMI) (32.2 kg/m^2^ versus 30.5 kg/m^2^; t=2.769, p=0.006). No difference in allergy predisposition (χ^2^=0.234) was noted. Tobacco use was significantly reduced in older patients (χ^2^<0.001). No differences in preoperative steroid medication (χ^2^=0.081) was found, but older patients were given non-steroidal anti-inflammatory drugs at a higher rate of time (χ^2^=0.010). Regarding comorbidities, a greater prevalence of cardiovascular diseases (χ^2^=0.001) in older patients existed, while no significant difference in respiratory diseases (χ^2^=0.104) or diabetes (χ^2^=0.109) was demonstrated. No significant difference in previous BMP exposure (4.0% vs. 3.4%, χ^2^=0.740) was elucidated.

Surgical treatment differed in both age groups. Patients older than 65 years underwent longer fusions (2.1 levels versus 1.7 levels, respectively, χ^2^<0.001), but no difference in total rhBMP-2 dose (12.5 mg versus 12.3 mg respectively; t=-0.934, p=0.351) or fusions utilizing more than one large kit (12 mg) rhBMP-2 (4.3% versus 2.8% respectively, χ^2^=0.369) was found. Elderly patients received lesser rhBMP-2 doses per level (7.0 mg versus 8.4 mg respectively; t=5.145, p<0.001).

Intraoperative diagnosed and repaired dural tears were more common in elderly patients (2.6% and 2.0% respectively; χ^2^<0.001). Revision surgery for clinically relevant nonunion was necessary in 3.6% of less than 65 year old patients compared to 3.9% in greater than or equal to 65 year old patients (χ^2^=0.872). A tendency to greater seroma formation was found in younger patients (4.4% versus 1.7% respectively, χ^2^=0.091). No significant difference in infection rate was diagnosed between the two age groups (χ^2^=0.857).

Comparing comorbidities as confounding factors for complications, no association between diabetes mellitus and infection (χ^2^=0.810), seroma formation (χ^2^=0.052), or nonunion (χ^2^=0.602) was found. Patients with existing respiratory disease did not show higher rates of infection, seroma formation, or nonunion (χ^2^=0.090, 0.974, and χ^2^=0.694 respectively). Patients with cardiovascular disease had an increase in seroma formation (χ^2^=0.041) but no differences for infection (χ^2^=0.062) or nonunion rate (χ^2^=0.0791).

## Discussion

Because of demographic changes and higher demands for mobility and life style, the number of elderly patients who seek help by orthopedic surgeons for lumbar pathology is increasing. In contrast to younger patients, degenerative lumbar pathology with stenosis is the leading cause for surgical intervention in the elderly [1]. Lumbar decompression and fusion can produce improvements in pain, ambulation, and activities of daily living [18] and therefore provides an independent lifestyle paramount to elderly patients. To achieve a solid fusion, stable instrumentation and a reasonable amount of osteoinductive material is required. The most commonly used fusion material is autologous iliac crest bone graft. Due to multilevel fusions requiring extensive osteoinductive material and poor bone quality void of osteoinductive material in elderly patients, a large amount of iliac crest bone graft is needed. Extensive graft harvest clearly lengthens the surgical procedure, increases blood loss [19], multiplies the chances of bone harvest related complications, and frequently is inadequate or impossible. To reduce the necessary amount of iliac crest autograft and enhance fusion, bone void fillers in combination with rhBMP-2 became a common supplement. Despite its off-label use, 30.0% of BMP use is in primary TLIF/PLIF procedures and 20.4% of BMP is used in primary posterolateral spinal fusions [20]. Hence, the use of BMP for lumbar spinal fusions can be considered as an optional “standard of care” before the FDA has granted approval [20].

The initial cost of rhBMP-2 remains an important concern. Possible cost reduction was attributed to fewer complications and decreased length of hospital stay [21]. Previous studies showed an average hospital stay for patients with lumbar spine fusion of 4 to 6 days [14,17,21]. In our study, average length of stay for all patients was 4.7 days. This is consistent with previous studies [14]. Even though not fully elucidate, rhBMP-2 in older patients may still be cost effective in reducing perioperative morbidity as a result of less donor site morbidity related to bone graft harvesting and non-health related costs [14,22]. Our study could not find a difference in hospital stay for patients who received only rhBMP-2 compared to those who had additional iliac crest bone graft harvest.

The efficacy of rhBMP-2 for posterolateral spine fusions has been demonstrated [12]. Reduction of rhBMP-2 efficacy in patients 65 years or older has been previously reported when compared to its efficacy in younger patients [6]. This might be related to differences in bone density or osteoinductive capacity [14]. We found no significant difference in nonunion rates comparing patients younger than 65 years of age to patients older than 65 years of age. Equal fusion rates could be attributed to a greater total BMP dose in older patients. Elderly patients in this study had more levels fused. Despite these factors resulting in a reduced BMP amount per level, no declination of BMP effectiveness or nonunion rates were noted.

Complication rates for lumbar spine surgeries included decompression and instrumentation for fusion in elderly patients differ from 7.1% [23] and 14.4% [3]. Our revision rate of 10.4% is within acceptable limits. Risks of operative procedures on the lumbar spine were reported to increase substantially with advancing age [3,18,24] but other studies showed no increased risk of complications in elderly patients [14,15,25]. This inconsistency may be caused by different age limits (65 years versus 70 years versus 75 years). In our study, the incidence of intraoperative dural tears was higher for patients greater than or equal to 65 years of age. This could be caused by increasing severity of stenosis due to degenerative changes in elderly patients as the cause for surgical intervention [3]. The most commonly reported complications related to rhBMP-2 in spinal fusions are osteolysis, edema, seroma, infection, and heterotopic bone formation [26-28]. We tried to elucidate the efficacy and risk profile for two age groups, but did not find significant differences between the groups. The reaction to rhBMP-2 still might be reduced in elderly patients due to the finding that seroma formation is less in patients older than 65 years of age.

No evident difference in nonunion rates or increased re-operation rates in both age groups suggests rhBMP-2 to be a potent enhancement irrespective of age. Further studies are warranted to elucidate the proper dosing and cost effectiveness.

The major limitation of this study was its retrospective design. The surgical outcome or complications were not compared to a contemporaneous control group and no differentiation regarding additional bone graft extenders could be performed. Small symptomatic seromas were counted in this study, but if they did not cause painful or neurological symptoms, they probably were not diagnosed or recognized as seromas. In this study, three seromas underwent percutaneous fluoroscopically guided aspiration. A higher number of small undiagnosed seromas with prolonged postoperative pain may be present. Radiculitis, as a complication attributed to rhBMP-2 utilization, has not been addressed in this study due to lack of practical tools to distinguish retrospectively between radiculitis and prolonged postoperative pain. Additionally, minor complications could be underrepresented or not recorded. We did not research clinical outcome, but no difference in clinical outcome rhBMP-2 versus ICBG in patients over 60 years of age has been found [7].

The strength of this study is the number of patients included. To our knowledge this study is the largest consecutive single center series with a homogeneous age distribution. All procedures were performed by five fellowship trained spine surgeons who have consistent treatment philosophies. Therefore, this study has the potential to reflect low incidence of complications.

## Conclusion

Increasing age has been reported a risk factor for major peri- and post-operative complications in patients undergoing lumbar spinal fusions. Complication rates in the elderly are high and it should be acknowledged that not all elderly patients will be suitable candidates for reconstructive fusion procedures. rhBMP-2 is a an effective bone graft replacement leading to successful posterolateral lumbar fusions, reduces the necessary amount of iliac crest bone graft procedures, and does not lead to increased complications in elderly patients.

## Competing interests

The authors do not have any interest that might be interpreted as influencing the research, and ethical standards were followed in the conduct and dissemination of the study. The authors did not receive grants or outside funding in support of their research or preparation of the manuscript.

## Authors’ contributions

MFH participated in the conception and design of the study, performed the data acquisition, participated in the statistical analysis, and drafted the manuscript. CBJ participated in the conception and design of the study, provided administrative support, carried out the critical revision of the manuscript and supervision of the study. DLS participated in the conception of the study, performed the statistical analysis, and critically revised the manuscript. All authors read and approved the final manuscript.
